# Maternal mortality in the Republic of Cyprus: Insights from population-based observations 2004–2022

**DOI:** 10.18332/ejm/212554

**Published:** 2025-12-19

**Authors:** Eleni Hadjigeorgiou, Vasiliki Piri, Antigoni Sarantaki, Maria Tigka, Athina Diamanti, Grigorios Karampas, Dimitra Metallinou

**Affiliations:** 1 Department of Nursing, School of Health Sciences, Cyprus University of Technology, Limassol, Cyprus; 2 Department of Midwifery, School of Health and Care Sciences, University of West Attica, Athens, Greece; 3 2nd Department of Obstetrics and Gynecology, Aretaieio University Hospital, Medical School, National and Kapodistrian University of Athens, Athens, Greece

**Keywords:** maternal mortality, Republic of Cyprus, epidemiological data, population-based

Maternal mortality remains a critical public health challenge, defined by the World Health Organization (WHO) as the annual number of deaths among women related to or aggravated by pregnancy, excluding accidental or incidental causes, occurring during pregnancy, childbirth, or within 42 days after its termination, regardless of the duration or anatomical location of the pregnancy^1,2^. To facilitate comparison across regions, the maternal mortality ratio (MMR) – the number of maternal deaths per 100000 live births – is widely used, as a key metric for monitoring progress and evaluating interventions^2^. While the International Classification of Diseases, Tenth Revision (ICD-10) limited maternal mortality statistics to deaths within 42 days postpartum, complications often extend beyond this timeframe, leading to the recognition of ‘late maternal deaths’ up to one year postpartum^1^.The updated ICD-11 now integrates both early and late maternal deaths into a unified category, improving the accuracy and consistency of maternal mortality reporting. This evolution in classification is vital for better understanding long-term pregnancyrelated risks and for guiding policies and resource allocation^1,3^. Addressing maternal mortality remains a global priority, with the United Nations Sustainable Development Goals (SDGs) targeting a reduction of the global MMR to fewer than 70 deaths per 100000 live births by 2030^4^.

This editorial marks the first effort to present comprehensive data on maternal mortality ratios and causes in the Republic of Cyprus, covering a period of nearly 20 years from 2004 to 2022. It explores how model-based estimates from global databases may differ from official national records, particularly in small-population countries. By identifying gaps in maternal healthcare and data collection, this editorial aims to inform policy improvements and enhance maternal health outcomes in the region.

## Global maternal mortality trends and Cyprus

According to WHO estimates, approximately 260000 women died in 2023 due to pregnancy-related complications, with a global MMR of 197 deaths per 100000 live births. While this reflects a 40% decline since 2000, significant disparities persist between high- and low-income countries^4^. In wealthier nations, maternal mortality remains exceptionally low, with an average of 10 deaths per 100000 live births, whereas low-income countries report a much higher ratio of 346 per 100000 in 2023^4^. In the Republic of Cyprus, the first official maternal mortality reports were recorded in 2004^5^, though these initial reports were limited to mortality rates.

We employed an epidemiological, descriptive, and retrospective design, analyzing all live births that occurred in the Republic of Cyprus during this timeframe. The study population included all pregnant women and those who gave birth in these areas, with follow-up extending up to one year after birth, regardless of nationality or residency status in Cyprus. Data collection involved systematic extraction of records from official sources, including the Cyprus Statistical Service, the European Statistical Office and the Ministry of Health. Where differences existed, records were reviewed to maximize completeness and accuracy. Data sources included medical death certificates, forensic and coroner’s reports, the national death register, hospital records, police and consular reports, occupational accident records, and data from the Cyprus national documentation and information center for drugs^5^. Causes of death were coded with ICD-10 using the WHO-endorsed IRIS software. Data entry and descriptive statistical analysis were performed using MS Excel, with annual live births serving as denominators for the calculation of maternal mortality ratios (MMRs). No data transformations or imputations were applied beyond the standardization of ICD-10 codes.

The findings revealed the birth trends for these years; annual live births fluctuated between 8309 and 10309. The data provide an overview of births by year, capturing the demographic trend over the period and provide the foundation for mortality analysis. The analysis of Table 1 shows an upward trend in births in the first period (2004– 2012), followed by a stabilization and slight decrease (2013–2019), and finally a new increase in the last years (2020–2022). During the 18-year period studied, 9 maternal deaths were recorded. The Republic of Cyprus achieved an exceptionally low maternal mortality ratio, often reaching zero in most years. The MMR peaked in 2014, reaching 21.6 deaths per 100000 live births, with two recorded maternal deaths that year (Table 1).

**Table 1:** Maternal mortality ratio (MMR) per 100000 live births and maternal deaths among live births in the Republic of Cyprus, 2004–2022

*Year*	*Births*	*Deaths*	*MMR*
2004	8309	0	0
2005	8243	0	0
2006	8731	1	11.45
2007	8575	0	0
2008	9205	1	10.86
2009	9608	0	0
2010	9801	1	10.20
2011	9622	0	0
2012	10161	1	9.84
2013	9341	1	10.71
2014	9258	2	21.6
2015	9170	0	0
2016	9455	0	0
2017	9229	1	10.84
2018	9329	0	0
2019	9548	0	0
2020	9930	0	0
2021	10309	0	0
2022	10197	1	9.81

Causes of maternal deaths included intrapartum and postpartum hemorrhage (O679, O721), severe preeclampsia (O141), threatened abortion (O200), obstetric blood-clot embolism (O882), ectopic pregnancy (O009), cardiac complications due to anesthesia (O291), and lastly other specified diseases and conditions complicating pregnancy, childbirth, and the puerperium (O998).

Furthermore, regarding the age distribution, most maternal deaths occurred in the 30–34 years age group (3 deaths). The 20–24 and 25–29 years age groups each accounted for 2 deaths. No deaths were recorded in the 15–19 and 45–49 years groups.

## Comparisons with other countries

The Republic of Cyprus, a European country with a population of 966365 in 2024, can be compared to other European nations with similar population sizes and geographical characteristics, such as Luxembourg (672050), Malta (563443), and Montenegro (623680)^6^. Maternal mortality, a key indicator of healthcare quality and access, remains relatively low across these countries. According to WHO data from 2023, Luxembourg reported 11.7 deaths per 100000 live births, Malta 8.4 and Montenegro 5.8^7^. These numbers indicate robust maternal healthcare systems, though minor fluctuations may result from variations in medical facilities, availability of maternity services, and governmental health regulations.

The WHO reports an MMR of 53 per 100000 live births for Cyprus in 2022^7^, a figure notably higher than the MMR calculated in our study using data from the Statistical Service and the Ministry of Health^5^. Given that the Republic of Cyprus has a well-developed healthcare system, with high prenatal care coverage and low maternal mortality rates, this discrepancy warrants further investigation. One key factor contributing to this difference is the WHO’s reliance on model-based estimates, particularly for countries with smaller populations where official data may be incomplete or inconsistent. These models incorporate regional trends and statistical adjustments, which can sometimes lead to an overestimation of maternal mortality, especially in countries like Cyprus with limited maternal mortality data. In contrast, our study is based on official national records^5^, which provide a direct and precise account of maternal deaths, making them a more accurate reflection of reality. Additionally, the WHO applies standardization techniques to ensure comparability across countries. While these adjustments are valuable for global monitoring, they may not always align with the specific healthcare context of Cyprus, where maternal health services are advanced, and mortality rates remain low. Another contributing factor is the time lag in international data collection and reporting. If the WHO’s estimate is based on outdated or incomplete figures, it may fail to accurately reflect the most recent maternal mortality trends in Cyprus. Furthermore, the WHO may aggregate MMR estimates for the Republic of Cyprus and the occupied northern part of Cyprus, rather than treating them separately. Since the healthcare systems, data collection methods, and maternal health outcomes differ between these two, this approach could introduce further discrepancies between the WHO estimate and the official national data of the Republic of Cyprus. Therefore, while WHO estimates are essential for global analyses, national data sources provide a more accurate and detailed representation of maternal mortality within Cyprus.

Importantly, the findings also highlight the role of midwives in sustaining low maternal mortality rates, as evidence shows that scaling up midwifery interventions could prevent the majority of maternal and neonatal deaths worldwide^8^. As frontline providers of maternity care, midwives play a critical role in the early recognition and management of complications, ensuring timely referrals, and supporting safe childbirth practices^9^. Their contributions to health education, preventive care, and systematic documentation further strengthen surveillance and reporting systems^10^. Global evidence further indicates that expansion of the nursing and midwifery workforce is significantly associated with reductions in maternal mortality, underscoring the strategic importance of investing in midwifery capacity^11^. In the Cypriot context, where maternal deaths are infrequent, midwives’ vigilance, continuity of care, and professional expertise are central to sustaining maternal health standards; however, as Kolokotroni et al.^12^ note, structural barriers constrain their educational and advocacy roles, underscoring the importance of fully utilizing midwifery capacity to further improve outcomes.

In conclusion, this editorial highlights the exceptionally low maternal mortality ratio (MMR) in the Republic of Cyprus from 2004 to 2022, demonstrating the effectiveness of its healthcare system and perinatal care. The discrepancy between WHO estimates and national data demonstrates the challenges of model-based projections in smallpopulation countries and underlines the importance of official records for accurate monitoring. Our findings emphasize the importance of using official national records for more accurate assessments of maternal deaths. Sustaining these achievements will require ongoing investment in robust data systems and continued strengthening of maternal health services. Crucially, empowering midwives to practice their full scope – by supporting their clinical, educational, and advocacy roles – will be key to maintaining low maternal mortality and further advancing maternal health in the Republic of Cyprus.

## Data Availability

Data sharing is not applicable to this article as no new data were created.
